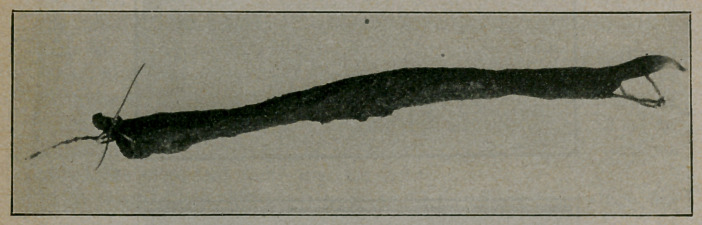# Cast of Uretha and Cicatrix Due to Silver Nitrate: Insanity

**Published:** 1913-01

**Authors:** 


Cast of Uretha and Cicatrix Due to Silver Nitrate: Insanity. Dr. Victor Cox Pedersen of N. Y., in the *Critic and Guide,* Nov., 1912, reports the case of a man who, expecting to marry and wishing to rid himself of gonorrhoea, asked a druggist for a bougie to burn out the urethra. A stick of silver nitrate was given him. The deep urethra was button-holed to obviate the resulting urinary obstruction. Under frequent irrigations of weak permaganate, sepsis was avoided and a cast of the anterior urethra, four inches long was passed in four days. Gonococci presisted in the discharge for some time. A dense but not impermeable stricture involved a corresponding length of the urethra. From disappointment at the breaking of his engagement and on account of syphilis, the man became insane. Dr. G. A. Smith, Supt. of the Central Islip State Hospital, reports the case as one of dementia praecox, of the paranoid type but states that, after eighteen months, urination is free without instrumentation. Through the courtesy of Dr. W. J. Robinson, Editor of the *Critic and Guide,* the accompanying illustration is reproduced.

## Figures and Tables

**Figure f1:**